# Editors’ Note

**DOI:** 10.5195/ijt.2016.6197

**Published:** 2016-07-01

**Authors:** ELLEN R. COHN, JANA CASON

## Abstract

The Spring 2016 issue of the International Journal of Telerehabilitation (IJT) presents original and innovative work in three diverse sections: usability, intervention, and pedagogy, followed by a book review on teleaudiology. The contributors to this issue are notably multi-disciplinary and include an audiologist, computer scientists, engineers, an epidemiologist, occupational therapists, a rehabilitation counselor, a physician (physical medicine and rehabilitation), and speechlanguage pathologists. The common thread linking the Journal’s authors and their manuscripts, is excellence in telerehabilitation related innovation.

One of the hallmarks of the peer-reviewed International Journal of Telerehabilitation (IJT) is the wide variety in the topics of the work presented, all of which relate to innovations in telerehabilitation. The Journal’s flexible format accommodates a variety of submission types: research studies, clinical reports, book reviews, country reports, and pedagogical notes. And, our authors and expert reviewers hail from multiple disciplines. It is especially gratifying that our contributors span the academic life cycle -- a mix of seasoned authors and new contributors. We are honored to serve as stewards of their work, and present it to our readers.

The Spring 2016 issue of the International Journal of Telerehabilitation (IJT) follows this tradition. Herein, we present original and innovative work in three diverse sections: usability, intervention, and pedagogy, followed by a book review on teleaudiology. The contributors to this issue are notably multi-disciplinary: an audiologist, computer scientists, engineers, an epidemiologist, occupational therapists, a rehabilitation counselor, physician/physical medicine and rehabilitation, and speech-language pathologists. A common thread linking all articles and authors, is excellence in telerehabilitation related innovation.

We applaud our publisher who facilitates the publication of IJT as an open source journal, with neither subscriptions, nor author fees. Our reviewers, editorial staff, and authors and their home institutions are similarly generous, so that current advances in the field of telerehabilitation can be widely disseminated.

## CALL FOR SUBMISSIONS

The next volume of the International Journal of Telerehabilitation will be published in Fall, 2016. We cordially invite your submissions by September 2, 2016. IJT accepts original research, case studies, viewpoints, technology reviews, book reviews, and country reports that detail the current status of telerehabilitation. Our peer reviewers constitute a multi-disciplinary group, and include researchers and clinicians from each of the major rehabilitation disciplines, rehabilitation engineers, health information managers, information technologists, and others. We welcome new peer-reviewers and invite guest editors with ideas for special, thematically focused issues. The IJT publication team is agile and can add additional issues as warranted to ensure currency. Please contact Editor Ellen Cohn, PhD (ecohn@pitt.edu) or Senior Associate Editor Jana Cason (jcason@spalding.edu) if you are interested in contributing to a future issue.

Respectfully,

Ellen R. Cohn, PhD, CCC-SLP

IJT Editor

Jana Cason, DHS, OTR/L, FAOTA

Issue Co-Editor and IJT Senior Associate Editor

